# Effectiveness of Facetectomy in Correction of Adolescent Idiopathic Scoliosis

**DOI:** 10.7759/cureus.55768

**Published:** 2024-03-08

**Authors:** Amjad Al Rashdan, Monther Alessa, Faris Ababneh, Faisal Al Taimeh, Zaid Althunaibat

**Affiliations:** 1 Department of Orthopedics, Royal Medical Services, Amman, JOR

**Keywords:** global coronal balance, curve cobb angle, posterior fusion, pedicle screws, facetectomy, adolescent idiopathic scoliosis

## Abstract

Introduction: Surgical correction of adolescent idiopathic scoliosis (AIS) using the facetectomy technique with the utilization of segmental pedicle screws aims to achieve correction of coronal and sagittal imbalances and preserve normal neurological function. In this study, we aimed to certify the effectiveness of the facetectomy technique in the correction of AIS by analyzing technique outcomes.

Methods: This is a retrospective, single-center study. From January 2018 to March 2022, a total of 51 patients with AIS who underwent inferior facetectomy with segmental pedicle screw constructs at the Royal Rehabilitation Center were reviewed. Radiological parameters including the major curve Cobb angle, and global coronal balance were evaluated preoperatively, postoperatively, and at the final follow-up. Surgical parameters and complications were also reported.

Results: The mean major curve Cobb angle was 59.5 ± 4.9° preoperatively, 13.6 ± 2.7° postoperatively, and 14.5 ± 2.6° at the final follow-up, with correction rates of 77.2% and 75.7%, respectively. The mean global coronal balance was 2.7 ± 1.1 cm preoperatively, 1.7 ± 0.73 cm postoperatively, and 1.4 ± 0.55 cm at the final follow-up. Two cases of pleural injuries were reported intraoperatively. Postoperatively, two cases experienced superficial wound infections, one experienced pulmonary embolism, and one patient had revision surgery due to the loosening of a single screw. None of these complications lasted long.

Conclusions: When combined with posterior segmental pedicle screw constructs, inferior facetectomy can provide an effective rate of correction in a reasonably safe manner.

## Introduction

The current literature reports an overall prevalence range between 0.47% and 5.2% for adolescent idiopathic scoliosis (AIS) making it the most prevalent form of scoliosis [1,2]. The prevalence of AIS can vary based on several factors such as gender, body mass index (BMI), familial history of scoliosis, and race or ethnicity [3-6]. Compared to males, females were found to have a higher prevalence [5,7,8]. It was obvious that the progression rate determined its eventual severity [9]. Untreated AIS might result in pulmonary dysfunction in rare cases indeed. However, most commonly, untreated AIS might result in severe deformity in adulthood, requiring deformity correction surgery. Pulmonary dysfunction most commonly occurs in the neuromuscular type of scoliosis [10]. Recently, posterior spinal fusion with segmental pedicle screw instrumentation has become the standard method for correcting and stabilizing the scoliotic curve [11-13]. In order to correct spinal abnormalities and restore flexibility, osteotomy is an essential procedure [14]. In the context of AIS, Ponte osteotomy is a common procedure for accomplishing rectification of major curvature [15-17]. Nonetheless, this technique potentially involves a higher risk of neural tissue injury and blood loss [16,18]. In contrast, adequate correction of the main AIS curves, acceptable thoracic kyphosis, less operative time, less blood loss, and less neural injury were reported in AIS patients treated with inferior facetectomies and segmental pedicle screws compared to Ponte osteotomies [19,20].

The purpose of this study was to demonstrate the effectiveness of the facetectomy technique in the correction of AIS by analyzing technique outcomes. We proposed that inferior facetectomies and segmental pedicle screw constructions would provide sufficient main curve rectification and satisfactory surgical results for AIS patients.

## Materials and methods

Study design and patients’ population

A retrospective study design was adopted to review patients’ files at the Royal Rehabilitation Center database/Jordan, where the spinal deformity corrections were routinely performed. All patients older than 10 years who suffered from AIS and were treated via the facetectomy technique from January 2018 to March 2022 were included in the study. Patients were classified according to Lenke et al., classification into one of the six main types [21]. In addition, patients were classified according to their skeletal maturity via the Risser classification system [22]. Patients were excluded if there was no six-month follow-up at least or if adequate radiographs were not available. In addition, patients’ electronic medical files that do not include the required data were excluded. Fifty-one patients met the inclusion criteria and were enrolled in the study.

Sampling technique

The patients’ data were gathered using a non-probability consecutive sampling method from those who adhered to eligibility criteria within the specified study duration.

Surgical procedure

**Figure 1:**
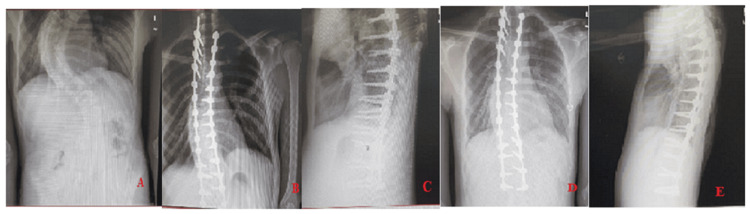
A 16-year-old male patient who underwent inferior facetectomies with pedicle screws instrumentation from T3-L4 levels. (A) preoperative posterior anterior (PA) x-rays, (B) immediate postoperative PA x-rays, (C) immediate postoperative lateral view x-rays, (D) follow-up after 12 months PA x-rays, and (E) follow-up after 12 months lateral view x-rays.

After the administration of general anesthesia and intubation, the patients were positioned prone with their abdomens suspended. Electromyography (EMG) monitoring was utilized and a midline paraspinal posterior incision was performed. The laminae, articular processes, and transverse processes were exposed after dissecting the subperiosteal tissues. Pedicle screws were inserted under (EMG) monitoring and C-arm imaging guidance into the chosen levels of fusion. To determine the fusion levels, shoulder balance was assessed (if right shoulder higher we start at T4, if left shoulder higher we start at T2, if leveled we start at T3) [21]. After placement of two screws in each level, we used EMG monitoring thresholds was utilized to stimulate them directly. To execute an inferior facetectomy (concave and convex), an osteotome was used to cut away a section of the inferior articular process from each individual vertebra in order to reveal the superior articular process of the lumbar spine. Following that, rods made of cobalt chrome or titanium were placed along the clips to progressively correct the scoliosis. Using local autograft and/or allograft bone, we decorticated the superior articular processes, laminae, and transverse processes at each vertebral level to facilitate a posterior fusion. Finally, a vancomycin powder wash was applied, and two drains were inserted (Figure 1A-1E). 

Data collection and outcome measures

Patient characteristics included age and gender. Furthermore, operation parameters and post-operative parameters, including operation time, number of fused levels, instrumented levels, hospital length of stay (LOS), and any in-hospital complications, were reviewed and recorded. The effectiveness of the facetectomy technique was determined via analysis of the radiological parameters and post-operative complications. Radiological parameters included the major curve Cobb angle and global coronal balance. The global coronal parameter was measured as the distance between the C7 plumb line and the central sacral vertical line (CSVL). Pseudoarthrosis radiographic assessment criteria were set by the loss of Cobb angle correction of more than 10 degrees and/or instrumentation failure. All of these parameters were reviewed at three time points: pre-operative, post-operative, and at the final follow-up after the operation.

Ethical consideration

All patients' data were handled with strict confidentiality, and the data were analyzed anonymously by patient ID number. No contact was made with patients or their relatives. This study was approved by the Ethics Review Board at Royal Medical Services under IRB number of 11-2022.​​​​

Sample size calculation

To achieve the study power, Slovin's formula for cohort studies was adopted. The population size for those who underwent facetectomy technique January 2018 to March 2022 was 59 patients. The Slovin’s formula based on population size and margin of error of 5.0% The minimum required sample size was 51 patients:

$n=N/(1+Ne^2 )$

n=59/1+59*0.0025

n=59/1+0.147=51

where N is the population size, e is the margin of error, and n is the sample size.

Statistical analysis

The statistical analyses were conducted using SPSS Version 28.0 (IBM Corp., Armonk, NY). To present sample characteristics, descriptive analysis in terms of mean, standard deviation, frequencies, and percentages were utilized. To examine the differences between preoperative, postoperative, and final follow-up radiography data, repeated-measures ANOVA (RM-ANOVA) was used. Moreover, the Mauchly's test of sphericity for equality of variances was carried out, and the Shapiro-Wilk test for the normality assumption was used to verify the normal distribution of scores across different points in time of the observation. However, both assumptions were satisfied. P<0.05 was considered statistically significant.

## Results

Fifty-one patients met the inclusion criteria of the study (Table 1); 36 females (70.6%) and 15 males (29.4%). At the time of surgery, the mean age was 15.3 ± 1.8 years (range, 12-18 years). The mean follow-up time was 14.4 months (range, 11-22 months). The mean Risser stage was 3.8 ± 1.7. Main thoracic (type 1) was the most frequent Lenke classification, presenting in 21 patients, followed by double major (type 3) in nine patients.

**Table 1:** Summary of patients’ demographics and (n=51) M: Mean, SD: Standard deviation, N: Number of cases, %: Percentage

Patient demographics	M ± SD
Age at time of surgery, years	15.3 ± 1.8
Risser stage	3.8 ± 1.7
Gender	N (%)
Male	15(29.4)
Female	36(70.6)
Lenke classifications, n (%)	
1.Main thoracic	21 (41.2)
2.Double thoracic	5 (9.8)
3.Double major	9 (17.6)
4.Triple major	6 (11.8)
5.Thoracolumber/lumber	6 (11.8)
6.Thoracolumber/lumber with main thoracic	4 (7.8)

The mean operative time was 5.2 ± 0.73 hours (range, 4-6.30 hours), with a mean number of fused levels of 11.3 ± 2.4 (range, 6-15 levels). Regarding the postoperation LOS in the hospital, the mean was 6.1 ± 2.1 days (range, 4-14 days) including the day of surgery. All patients were discharged from the operating room with two surgical drains. The average postoperative blood loss was 480 ± 280.3 mL (range, 100-1,230 mL) (Table 2).

**Table 2:** Summary of patients’ operative data (n=51) M: Mean, SD: Standard deviation

Operative data	M±SD
Operative time, hours	5.2 ± 0.73
Fused levels	11.3 ± 2.4
Postoperation hospital stay, days	6.1 ± 2.1
Postoperation blood loss, mL	480 ± 280.3

Regarding intra-operative complications, two patients experienced pleural injury, which was treated by the thoracic surgeon during surgery via stitches; one of them developed pneumothorax, and the other one developed hemothorax (chest tubes drainage was inserted for both of them during surgery). Two patients developed superficial surgical site infections following surgery, which were treated with appropriate dressings and antibiotics. One patient developed a pulmonary embolism and was admitted to the intensive care unit on postoperative day. The patient was treated with warfarin treatment for three months and followed by a pulmonary physician. Two patients required revision surgery, one of whom experienced loss of signal during surgery and the other of whom required the replacement of one of the pedicle screws. No other patients had any additional serious complications, such as neurological deficiency. No pseudoarthrosis cases were reported after surgery (Table 3).

**Table 3:** Summary of intra-operative and postoperative complications N: Number of cases, %: Percentage

Intra-operative complications	N (%)
Pleural injury	2 (3.92)
Loss of signal and aborted operation	1 (1.96)
Post-operative complications	
1.Superficial wound infections	2 (3.92)
2.Pulmonary embolism	1 (1.96)
3.Repeated surgery	2 (3.92)

The mean preoperative major curve Cobb angles were 59.5 ± 4.9 degrees. The mean of the major curve Cobb angle was 13.6 ± 2.7 degrees postoperatively and 14.5 ± 2.6 degrees at final follow-up, for which the correction rate was 77.2% and 75.7%, respectively. Postoperatively, there was a significant improvement in the major curves of Cobb angle (P<0.001). The mean global coronal balance was 2.7 ± 1.1 cm preoperatively, 1.7 ± 0.73 cm postoperatively, and 1.4 ± 0.55 cm at the final follow-up. The global coronal balance improved significantly postoperatively and at the final follow-up (P<0.001) (Table 4).

**Table 4:** Preoperative, postoperative, and final follow-up of radiologic measurements P-value 1: compared between pre-op and post-op P-value 2: compared between post-op and final follow-up *Bonferroni adjustment was taken into consideration for pairwise comparisons

Radiologic parameters	Pre-op	Post-op	P-value 1*	Final follow-up	P-value 2*
	Mean±SD	Mean±SD		Mean±SD	
Major curve Cobb angle, °	59.5 ± 4.9	13.6 ± 2.7	< .001	14.5 ± 2.6	< .001
Global coronal balance, cm	2.7 ± 1.1	1.7 ± 0.73	< .001	1.4 ± 0.55	< .001

## Discussion

AIS is a complicated, three-dimensional (3D) deformity of the spine and rib cage [23,24] characterized by a lateral curvature of the spine in the coronal plane that is greater than 10 degrees [25]. Treatment strategies, either conservative or surgical, aim to achieve correction of the scoliotic curve and prevent further progression. However, the degree of scoliosis plays a critical role in determining the treatment approach [26]. Conservative treatment is recommended for patients with a Cobb angle of fewer than 50 degrees. Otherwise, surgical treatment is recommended for patients with curves measuring greater than 50 degrees [27]. In literature, the majority of patients with Risser stage zero are still growing with progressive curves between 20 and 45 degrees and may have their curves arrested with a well-designed and adapted brace giving a 50% correction [28]. In the presented study, all patients had Cobb angles greater than 50 degrees. As a result, surgical treatment was decided. Hibbs [29] pioneered the surgical method, and since then, other modifications to the technique have been proposed, such as posterior [30] and anterior spinal fusion [31] followed by the development of segmental hook instrumentation, segmental pedicle screw constructions, and hybrid constructs [32].

Posterior segmental pedicle screw instrumentation and fusion is one of the most common surgical techniques utilized for scoliosis correction in AIS patients due to a lower complication rate [33,34]. As a result, SDRRT is mostly commonly performed. In this technique, two rods were connected to the heads of screws to facilitate rotation and correction of scoliosis [35-37]. Postoperative thoracic kyphosis might also be affected by the curvature of the implant rods. Sagittal alignment depends on the rod's original form. It is nonetheless well known that rods that have been pre-bent by surgeons before implantation have a tendency to straighten out after implantation [38,39]. Spinal sagittal alignment and clinical success may be affected by the “spring-back” impact of implant rod deformation after surgery.

Facetectomy is a technique utilized to facilitate bone fusion and make spinal deformities flexible. In the current study, facetectomy with a segmental pedicle screw was utilized instead of a Ponte osteotomy for the correction of scoliosis. Ponte osteotomies carry a risk of neural tissue injury and blood loss [16,18]. In our case series, the mean major curve Cobb angle of AIS patients was 59.5° and improved post-operatively to 13.6° and 14.5° at the final follow-up, with a correction rate of 77.2% and 75.7%, respectively. Compared to previous studies [15,20], our study achieved a higher correction rate than Samdani et al.'s study (67%) [15] and a lower correction rate than Halanski and Cassidy's study (83%) [20]. Our results provide some support for the hypothesis that inferior facetectomies in conjunction with segmental pedicle screw constructions may effectively correct coronal deformities.

During deformity correction with inferior facetectomies, it is crucial to properly position pedicle screws. Pedicle screws experience more biomechanical stress during corrective manipulation when compared to Ponte osteotomies because inferior facetectomies do not give as much of a posterior release. In our study, all pedicle screws were placed under C-arm fluoroscopic guidance.

Postoperatively, the global coronal balance was 1.7 cm, and at the end of follow-up, it was 1.4 cm. These findings show that the spine's balance has been restored to an acceptable degree. Similar results were also reported in the Elnady et al.'s study [40].

This is consistent with a normal thoracic kyphosis and lumbar lordosis range and demonstrates the ability of all pedicle screws in combination with facetectomy using the posterior technique to preserve normal sagittal alignment [19,41]. In our practice, we addressed both the sagittal and coronal components of kyphosis in the thoracic spine. 

Due to the serious risks involved with AIS surgery, including prolonged operational time, heavy blood loss, and catastrophic consequences such as spinal cord injury, patient safety must take precedence over deformity correction. The mean operational time in our study was 5.2 hours, which lies within the range reported in previous studies of 4.61 to 5.35 hours [15,17]. Intraoperative blood loss could not be assessed due to the unavailability of the required data in patient medical files. However, the mean postoperative blood loss was 480 mL. There were only a few complications in this series, with minimal duration, with the exception of one patient who had a pulmonary embolism and was treated with warfarin for three months until recovery. The prevalence of postoperative superficial surgical wound infection was minimal with two patients (3.92%) showing proximal wound disintegration. The wounds were successfully healed on antibiotic treatment with no surgical debridement required and wound cultures were negative for both cases. Our findings were aligned with Smith et al. who reported the rate of infection after spine surgery as 2.1% in a total of 108,419 cases [42]. After surgery, all AIS patients were neurologically evaluated, and no neurological deficits were detected post-operatively or at the final follow-up.

 In terms of implant-related complications, the loosening of a single screw necessitated revision surgery for an AIS patient. Implant dislodgment has been reported to be responsible for between 0.64% and 1.37% of complications in AIS spinal surgeries [43]. In terms of pulmonary complications, two patients had pleural injuries that were treated during surgery, and chest tubes were placed. One patient suffered from a hemothorax, while the other suffered from a pneumothorax. The duration for the chest tube was seven days for both patients. The amount of drainage was 650 mL for pneumothorax patients and 800 mL for hemothorax patients. In the Liang et al.'s report, 24 cases of hemothorax were found in the 3,325 AIS patients who underwent spinal deformity correction surgery [44].

However, there are a few limitations to this study. Because of the absence of a comparable control group, we were unable to compare the effectiveness of facetectomies to other procedures, such as Ponte osteotomies. Hence, previous studies presented heterogeneous data about the effectiveness of the aforementioned procedures [15,20]. Furthermore, our study was retrospective, had a small sample size, and some variables, such as BMI and blood loss during surgery, were not included in the study due to their unavailability in the patient's medical file. However, given that the average follow-up period was 14.4 months, we are satisfied that this time frame was adequate to answer the questions addressed by the current study. In spite of these limitations, we are convinced that the results of our investigation will provide valuable insight into the limited literature on this method.

## Conclusions

For patients with AIS and poor flexibility, the facetectomy procedure, combined with posterior segmental pedicle screw constructs, can provide an effective rate of correction of the main curve Cobb angle, coronal, and sagittal plane alignments. The technique was relatively safe, but it is essential that surgeons who treat AIS patients be familiar with the nature and treatment of potential complications.
